# Pre-emptive Laparoscopic Colostomy Creation in Obstructing Locally Advanced Rectal and Anal Cancer Does Not Delay the Starting of Oncological Treatments

**DOI:** 10.3390/cancers16162799

**Published:** 2024-08-08

**Authors:** Giovanni Taffurelli, Isacco Montroni, Claudia Dileo, Alessandra Boccaccino, Federico Ghignone, Davide Zattoni, Giacomo Frascaroli, Giampaolo Ugolini

**Affiliations:** 1 Colorectal and General Surgery Unit, Ospedale Santa Maria delle Croci—AUSL Romagna, 48121 Ravenna, Italy; 2 Dipartimento Scienze Mediche e Chirurgiche (DIMEC), University of Bologna, 40126 Bologna, Italy; 3 Medical Oncology Unit, Ospedale Santa Maria delle Croci—AUSL Romagna, 48121 Ravenna, Italy

**Keywords:** rectal cancer, anal cancer, occlusion, colostomy, neoadjuvant therapy

## Abstract

**Simple Summary:**

We conducted an evaluation of the outcomes associated with pre-emptive laparoscopic colostomy in patients suffering from obstructing rectal and anal cancer. Our findings reveal that this surgical approach has a role in facilitating the timely initiation of therapy, without causing significant delays. The ability to start treatment promptly is particularly important for these patients, as they often face advanced stages of disease and significant nutritional challenges. Our study supports the viability of this surgical method as an effective strategy for managing obstructing cancers, ensuring that patients receive the necessary treatment as quickly as possible. By addressing the obstruction early, this approach not only improves patient outcomes but also enhances their overall quality of care during a critical time.

**Abstract:**

Background: Managing patients with obstructing rectal cancer is challenging due to the risks of gastrointestinal obstruction and perforation. This study evaluates the outcomes of pre-emptive laparoscopic colostomy creation in patients with locally advanced rectal and anal cancer to prevent symptoms and facilitate therapy initiation. Methods: This retrospective cohort study includes patients with locally advanced rectal or anal cancer assessed by our Colorectal Multidisciplinary Team from January 2017 to February 2024. Patients who underwent pre-emptive laparoscopic colostomy were compared to a control group of non-obstructing rectal cancer patients who started direct oncological treatment. The primary endpoint was the time from diagnosis to the initiation of oncological treatments. The secondary endpoints were the rate and timing of subsequent radical resection, surgical morbidity and hospital stay. A Weibull regression was used to evaluate the time differences between the groups. Results: There were 37 patients who received pre-emptive laparoscopic colostomy, compared to 207 control patients. The mean time from diagnosis to the start of neoadjuvant therapy was 38.3 ± 2.3 days. Despite higher rates of malnutrition and more advanced stages in the colostomy group, no significant differences were observed in the time to start therapy (*p* = 0.083) or time to radical resection (*p* = 0.187) between the groups. The laparoscopic procedure showed low rates of postoperative complications and acceptable lengths of stay. Discussion and Conclusions: Pre-emptive laparoscopic colostomy is a feasible approach for managing obstructing rectal or anal cancer. Treatment timelines were not extended compared to timelines for non-obstructing cases, despite differences in nutritional status and staging. Further prospective studies with larger cohorts are needed to validate these findings and refine treatment protocols for obstructing gastrointestinal malignancies.

## 1. Introduction

The landscape of neoadjuvant treatment for locally advanced rectal cancer has recently expanded with different therapeutic strategies based on patient and tumor factors. A major concern for medical/radiation oncologists and surgeons is managing patients with obstructing ano-rectal cancer. Evidence shows that patients undergoing radiation therapy have twice the risk of hospital admission due to gastrointestinal issues, and up to 10% may experience high-grade bowel obstruction [1,2,3]. The lack of pre-emptive behavior and the threat of emergency surgery poses a substantial risk for these patients. It has been demonstrated that patients treated in an emergency setting experience significant delays in resuming their treatments and suffer from stoma malposition and related complications in up to one-third of cases [4]. Furthermore, patients with anal cancer experience not only a high risk of obstruction but also a greater chance of fistulas to other pelvic organs and perianal sepsis, highlighting the need for a pretreatment stoma [5].

Some studies have explored the topic of a diverting colostomy in obstructing/near obstructing rectal cancer, concluding that patients requiring this intervention often face significant delays in resuming their oncological treatments. However, these studies varied in their patient population for the following reasons: some included patients who underwent emergency surgery due to complete large bowel occlusion, others involved a laparotomic approach which caused surgery-related delays, and some involved patients who proceeded directly to upfront surgery instead of proceeding with neoadjuvant treatments [6,7,8].

The goal of this study was to evaluate the surgical outcomes of patients undergoing pre-emptive laparoscopic colostomy for locally advanced rectal and anal cancer in a non-urgent/emergent setting, aiming to prevent occlusion and to allow the timely initiation of therapies. The group was compared to a control group of non-obstructing locally advanced rectal cancer patients to compare the length of time needed to start oncological therapy between the two groups.

## 2. Methods

This study is a retrospective cohort study derived from a prospectively maintained database, approved by the Institutional Review Board as part of the Ravenna Surgical Quality Improvement Program (RaSQIP). All consecutive patients evaluated for locally advanced rectal or anal cancer by our Colorectal Multidisciplinary Team (MDT) from January 2017 to February 2024 were included. Additionally, resectable oligometastatic patients recommended for neoadjuvant therapy by the MDT, aiming for a subsequent R0 resection, were also included.

At our institution, the indication for pre-emptive colostomy placement is considered for every patient with obstructing rectal cancer and incomplete colonoscopy, as well as for every patient with perforated cancer. The timing of colostomy creation is carefully evaluated on a case-by-case basis, especially as pelvic-related or sub-occlusive symptoms worsen, to prevent high-grade bowel obstruction. A laparoscopic approach is offered to all patients, and the procedure of choice is a loop colostomy at the level of the sigmoid colon in the case of impending obstruction, while an end colostomy is performed in the case of fistulizing cancers. Although the literature describes transverse colostomy as a valid option for managing bowel obstruction due to colorectal cancer, we believe that using the sigmoid colon may offer several advantages. Transverse colostomy is primarily recommended for sigmoid or left obstructing colon cancer to avoid colonic stenting [9]. It is mainly employed in emergency settings and typically performed using an open approach. In our experience, since the colostomy creation is pre-emptive and intended to prevent complete bowel obstruction, using the sigmoid colon is simpler. The sigmoid loop is often long enough to easily reach the abdominal wall laparoscopically, requiring minimal mobilization. An additional advantage of utilizing the sigmoid colon is evident in cases where patients have completed neoadjuvant therapy and are scheduled for formal rectal resection. In these situations, the site of the colostomy can often be used as the lower margin for the resection, serving as a convenient point for creating a colo-anal anastomosis. Alternatively, it can be converted to an end colostomy if an abdomino-perineal excision is needed. Furthermore, compared to transverse colostomy, the sigmoid colostomy likely facilitates easier endoscopic colonic surveillance, if required, especially given the presence of obstructing rectal cancer.

Exclusion criteria for the study were the following: Stage 1 rectal cancer, as these patients benefit from primary surgery; palliative colostomy in patients with extensive metastatic disease who received only palliative treatments right after; and patients who underwent emergency surgery where rectal cancer was diagnosed following a large bowel obstruction.

**Figure 1:**
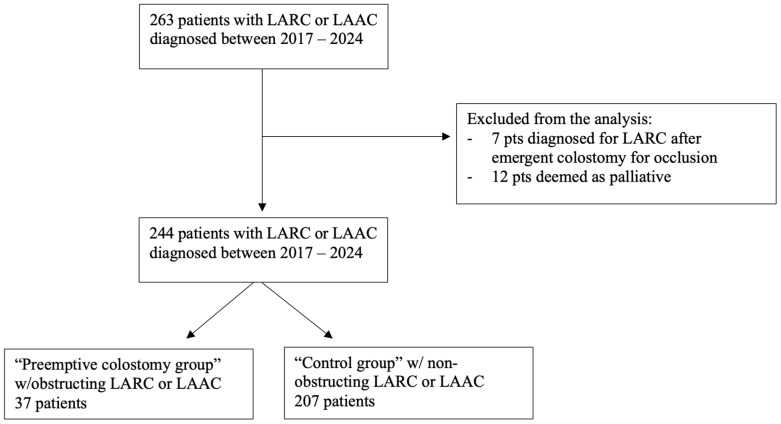
Flowchart of patients’ selection. Legend: LARC = locally advanced rectal cancer. LAAC = Locally Advanced Anal Cancer.

Contraindications essentially reflect the exclusion criteria, particularly for patients deemed suitable only for palliative treatment or supportive care. At our institution, a past medical history of abdominal surgery is not considered a contraindication to offer a laparoscopic approach. The patients’ selection flowchart is shown in Figure 1.

Demographic data such as sex, age, Eastern Cooperative Oncology Group–Performance Status (ECOG-PS) [10], and the Nutritional Risk score (NRS-2002) [11] derived from the European Society for Clinical Nutrition (ESPEN) guidelines were collected. Oncological parameters such as tumor site, distance from the anal verge, staging according to AJCC 8th edition for rectal cancer and 9th edition for anal cancer [12,13], and type of neoadjuvant therapy were evaluated. For the surgical group, additional data such as BMI, Charlson Comorbidity Index (CCI) [14], American Society of Anesthesiologists (ASA) score, type of surgical approach, time from diagnosis to colostomy placement, time from colostomy to therapy, and time from initial diagnosis to therapy start were recorded.

The postoperative course was classified at the time of discharge, and complications up to 90 days post-discharge were recorded and categorized using the Clavien-Dindo Classification (CDC) [15]. Comprehensive Complication Index (CCI^®^) scores [16] were calculated from all postoperative complications using an online calculator. The length of stay (LOS) was defined as the time from the day of surgery to discharge.

The pre-emptive colostomy group was compared to a control group of patients for whom a neoadjuvant therapy was indicated and completed during the study period. This comparison aimed to evaluate the impact of the surgical intervention on potential delays in the standard timeline for initiating oncological treatments. The primary endpoint was the difference in time from diagnosis to the start of the oncological therapy. The secondary endpoints included the rate and timing of radical resection after neoadjuvant treatments, postoperative morbidity/mortality and length of stay after stoma creation.

Regarding the timing of radical resection following neoadjuvant treatments, resection rates pertain exclusively to patients who, after undergoing neoadjuvant therapy, had a formal indication for surgical resection. Patients with either anal or rectal cancer who achieved a complete response were excluded from the resection rate analysis in both groups.

### Statistical Analysis

Data were reported as percentages or means and standard deviations (SDs). The two groups were compared for demographic factors, tumor-related factors, and time from diagnosis to therapy. Differences between the groups were measured using Student’s *T*-test and Fisher’s Chi-Square test, considering a *p* value < 0.05 as statistically significant. The Akaike Information Criterion (AIC) and Bayesian Information Criterion (BIC) were used for probabilistic model selection and goodness of fit [17]. A Weibull regression was constructed to evaluate differences in the timing of starting oncological therapy and radical resection between the two groups. Statistical analysis was performed using STATA software Version 18 (StataCorp. 2017. College Station, TX: StataCorp LP).

## 3. Results

The demographic and surgical characteristics of the colostomy group are reported in Table 1. Of the 244 patients evaluated at our institution for locally advanced rectal or anal cancer, 37 patients (15.1%) underwent a loop colostomy for obstructing/symptomatic rectal or anal cancer before starting any oncological treatment. Most patients were male (21; 56.8%), with a mean age of 68.9 ± 2.2 years, and almost 80% had rectal cancer. The mean distance from the anal verge was 7 cm. The vast majority had an ECOG-PS score of 0 or 1 (35; 94.6%), while a high rate of malnutrition was observed (NRS 2-3 in 40.5% of patients). Cancer staging reflected advanced tumors, with 32% being oligometastatic and 56.8% at Stage 3. The four Stage 2 patients were all affected by squamous cell carcinoma of the anus. All patients were treated laparoscopically (37; 100%). Only one patient (2.7%) experienced a severe postoperative complication (CDC 3a). The mean CCI for the entire group was 9.0 ± 2.3%, and the mean length of stay was 4.1 ± 0.8 days, with a readmission rate of 5.4% (2 patients) and no need for reoperation. The mean time from endoscopy to colostomy placement was 16.7 ± 11 days, and the mean time from surgery to the start of any neoadjuvant therapy was 23.4 ± 1.8 days. Overall, the mean time from diagnosis to the start of any neoadjuvant therapy, including surgery for colostomy creation, was 38.3 ± 2.3 days. Twenty-one patients (61.7%) underwent radical resection after neoadjuvant therapy (three patients are currently undergoing oncologic treatments).

The comparison between the colostomy and control groups is reported in Table 2. The two groups were comparable in terms of sex, age, ECOG-PS, cancer site, and distance from the anal verge. However, they differed in nutritional scores (*p* = 0.002) and staging, with a higher prevalence of oligometastatic cancers in the colostomy group (32% versus 4%; *p* < 0.001). Consequently, the neoadjuvant strategies that were planned for these patients were significantly different (*p* = 0.016). A greater number of patients in the control group achieved radical resection after therapy (97% versus 61.7%; *p* = 0.021). For the primary endpoint, no statistical differences were observed in the time from diagnosis to the start of any neoadjuvant therapy between the two groups (38.3 ± 14.3 days in the colostomy group versus 33.5 ± 14.9 days for the control group; *p* = 0.083). In relation to the secondary endpoint, no differences were found in the time to radical resection for the patients who completed their therapies in the colostomy and control groups (7.8 ± 0.8 months versus 6.5 ± 0.3 months, respectively; *p* = 0.187).

**Figure 2:**
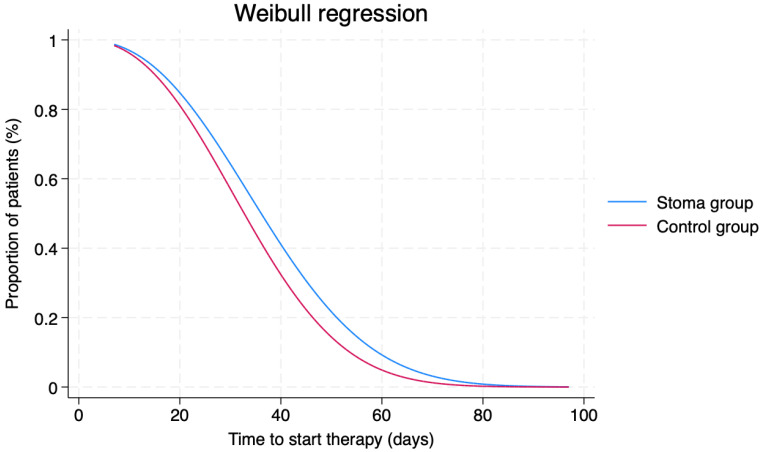
Weibull regression to evaluate differences in the timing of starting oncological therapy between the two groups. *p* = 0.184.

**Figure 3:**
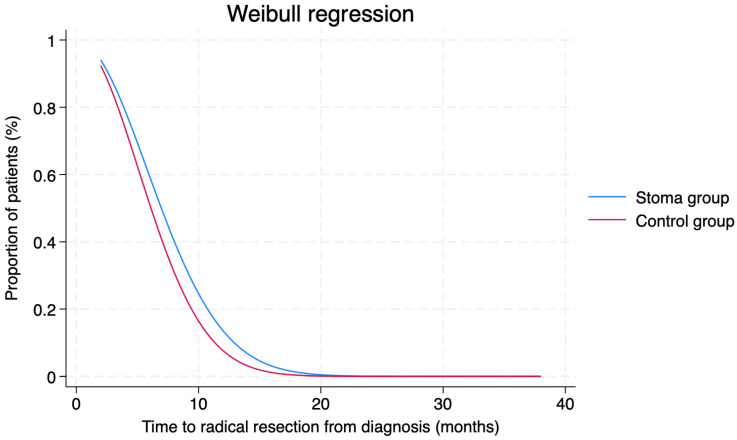
Weibull regression to evaluate differences in the timing of radical resection between the two groups. *p* = 0.352.

These results were confirmed by Weibull regressions, which indicated no significant differences between the two groups in the probability distribution regarding the time to start therapy (*p* = 0.184; Figure 2) and the time to achieve radical resection (*p* = 0.352; Figure 3).

## 4. Discussion and Conclusions

Various studies have explored the role of pre-emptive diversion in obstructing rectal cancer, highlighting the potential for significant treatment delays. However, discrepancies in patient cohorts across studies, including those undergoing emergency surgery, laparotomic approaches or upfront surgery, contribute to the inability to interpret data and standardize a common approach for these patients [6,7,8].

Our study aimed to evaluate the surgical outcomes of pre-emptive laparoscopic colostomy in patients with locally advanced rectal and anal cancer, aiming to mitigate the risk of occlusion and facilitate timely therapy initiation. By comparing a colostomy group to a control group of non-obstructing locally advanced rectal cancer patients, we sought to identify differences in therapy initiation timing.

Our findings suggest that pre-emptive laparoscopic colostomy is a safe and feasible approach for managing obstructing or symptomatic rectal or anal cancer, allowing for timely therapy initiation. Despite the complexity of these cases, characterized by advanced disease stages and significant nutritional risks, the laparoscopic procedure was associated with low rates of severe postoperative complications and acceptable lengths of stay.

Although the colostomy group exhibited differences in nutritional status and staging compared to the control group, there were no significant disparities in therapy initiation timing or time to achieve radical resection. These findings suggest that pre-emptive colostomy does not unduly delay treatment timelines for patients with locally advanced rectal or anal cancer. These data sharply contrast with the previous literature, especially the work by Patel et al. [6]. In that study, the authors attempted to simulate a “randomized trial” by dividing obstructing rectal cancer patients into the following two groups: diverted and non-diverted, with the latter receiving neoadjuvant treatments directly. However, this methodology introduces several significant biases, particularly in patient selection and management. For instance, the indication to perform a pre-emptive diverting colostomy, which is the case in nearly half of the study population, is not clearly stated. Additionally, the authors underestimated that nearly 10% of initially non-diverted patients required a diversion during the neoadjuvant therapy. Based on this analysis, we decided to compare our pre-emptively diverted group with a control group of asymptomatic patients. We believe this approach could provide a clearer understanding of the true impact of adding a colostomy during standard neoadjuvant treatment pathways, which could also benefit the non-negligible percentage of locally advanced rectal cancer patients who experience progression under neoadjuvant therapy [18].

A greater number of patients in the control group were able to undergo radical resection after therapy (97% versus 61.7%; *p* = 0.021). This difference is mainly explained by the fact that almost one-third of the patients undergoing a pre-emptive colostomy had metastatic disease and experienced cancer progression during the study period. 

It is crucial to recognize several limitations in our study. First, being a retrospective cohort study, it is susceptible to inherent biases and confounding factors that might impact the observed outcomes. Second, the sample size of the colostomy group may have been relatively small, potentially restricting the generalizability of our findings. Another inherent limitation of this study is the lack of an analysis on both short- and long-term quality of life (QoL) data, particularly in patients who underwent pre-emptive colostomy. Although some studies suggested a poorer quality of life for patients with stomas [19], others pointed out that QoL in patients with stomas is not inferior to patients who underwent a restorative procedure and then experienced major low anterior resection syndrome (LARS) [20]. It is important to point out that LARS is highly prevalent following restorative proctectomy and can affect up to 70–90% of patients [21,22].

Moreover, a recent large prospective multicenter study found no significant differences in QoL scores at 3 and 6 months in patients undergoing a low anterior resection or abdomino-perineal resections with permanent colostomy [23]. These findings further support our hypothesis that a preventive stoma creation should only partially decrease QoL while allowing prompt initiation of oncological treatments, with the ultimate goal of achieving radical tumor resection.

Although a QoL analysis would have provided valuable insights into the patient experience with a colostomy, it was not feasible with our current dataset. Our data lacked the comprehensive, longitudinal QoL assessments necessary for robust conclusions. Future research incorporating detailed QoL measurements is essential to gain a deeper understanding of the impact of pre-emptive colostomy on patient well-being.

Lastly, it is worth noting that some authors in the literature have explored the benefits of laparoscopic ileostomy creation in cases of obstructing rectal and distal colon cancer [24]. While postoperative results indicate that laparoscopic ileostomy creation can be a safe and feasible procedure, it is well documented that the presence of a loop ileostomy can increase the length of stay (LOS), hospital readmission rates, and the number of emergency department visits. Consequently, this procedure can lead to increased hospital costs and reduced time at home [25]. Additionally, the negative effects of ileostomy creation, such as electrolyte imbalance, renal failure, and ileus, have been well described. These complications are primarily related to dehydration, which is the most common cause of postoperative readmission, occurring in 17-21% of cases [26]. Recently, the role of transverse colostomy has also been questioned by some authors, who have reported a higher incidence of stoma prolapse when using the transverse colon compared to the sigmoid colon [27].

Our study contributes to the growing body of evidence supporting the role of pre-emptive sigmoid colostomy in optimizing the management of obstructing rectal cancer, emphasizing the importance of individualized treatment strategies. Additional prospective studies with larger cohorts and extended follow-up periods are necessary to validate these findings and enhance treatment algorithms for patients with locally advanced rectal and anal cancer. Furthermore, the emergence of new drugs and the identification of novel molecular pathways have the potential to revolutionize the management and treatment response of these patients.

## 5. Conclusions

In summary, our study highlights the role of pre-emptive laparoscopic colostomy in the surgical management of obstructing rectal and anal cancers. This approach has been an effective strategy, enabling timely initiation of therapy without significant delays. Despite variations in nutritional status and disease staging, patients who underwent pre-emptive colostomy did not experience prolonged treatment timelines compared to those with non-obstructing cases.

Looking ahead, refining patient selection criteria and optimizing treatment protocols will further enhance the benefits of pre-emptive colostomy in cases of locally advanced rectal and anal cancer. There remains a delicate balance between near-obstructed and fully obstructed patients, and current predictive models are insufficient for accurately forecasting adverse outcomes. Prospective studies with larger cohorts and extended follow-ups are needed to validate these findings and develop evidence-based guidelines for managing obstructing gastrointestinal malignancies.

## Figures and Tables

**Table 1:** Characteristics of 37 patients undergoing pre-emptive loop colostomy for obstructing rectal or anal cancer.

Characteristics	*N*(%) or Mean (SD)
**Sex**	
Male	21 (56.8)
Female	16 (43.2)
**Age**	68.9 ± 2.2
**BMI** (kg/m^2^)	23.5 ± 0.6
**CACI**	6 ± 0.5
**ASA**	
1	2 (5.4)
2	16 (43.2)
3	17 (45.9)
4	2 (5.4)
**Type of cancer**	
Rectal cancer (Adenocarcinoma)	29 (78.4)
Anal cancer (Squamous cell carcinoma)	8 (21.6)
**Distance from AV (cm)**	7.2 ± 0.8
**ECOG-PS**	
0–1	35 (94.6)
2–3	2 (5.4)
**NRS**	
0–1	22 (59.5)
2–3	15 (40.5)
**Therapy after colostomy**	
LC-RT	10 (27.0)
TNT	15 (40.5)
First-line chemotherapy	12 (32.5)
**Stage according to AJCC**	
2	4 (10.8)
3	21 (56.8)
4	12 (32.4)
**Time from diagnosis to colostomy placement** (days)	16.7 ± 11.1
**Starting therapy from colostomy** (days)	23.4 ± 1.8
**Starting therapy from diagnosis** (days)	38.3 ± 2.3
**Laparoscopic approach**	37 (100)
**Any postoperative complications**	14 (37.8)
**Clavien Dindo**	
0	23 (62.2)
1	3 (8.1)
2	10 (27.0)
3	1 (2.7)
**CCI** (%)	9.0 ± 2.3
**Length of stay** (days)	4.1 ± 0.8
**Readmission**	2 (5.4)
**Reoperation**	0 (0)
Radical resection rate after therapy or resection not needed *^?^	21/34 * (61.7)

Legend: BMI: Body Mass Index; CACI: Age-Adjusted Charlson Comorbidity Index; ASA: American Society of Anesthesiology score; AV: anal verge; ECOG-PS: Eastern Cooperative Oncology Group–Performance Status scale; NRS: Nutrition Risk Screening score; LC-RT: long course chemio-radiotherapy; TNT: Total Neoadjuvant Therapy; AJCC: American Joint Committee on Cancer; CCI: Comprehensive Complication Index; * 3 patients still ongoing therapy; ^?^ 6 patients did not undergo radical resection for complete clinical response after therapy.

**Table 2:** Comparison of 37 patients undergoing pre-emptive loop colostomy with a similar cohort of 207 patients with locally advanced non-obstructing rectal/anal cancer.

Characteristics	Diverting Colostomy Group	Non-Obstructing Group	*p* Value
**Sex**			
Male	21 (56.8)	108 (52.2)	0.607
Female	16 (43.2)	99 (47.8)	
**Age** (mean ± SD)	68.9 ± 2.2	67.7 ± 0.8	0.579
**ECOG-PS**			
0–1	35 (94.6)	189 (91.3)	0.502
2–3	2 (5.4)	18 (8.7)	
**NRS**			
0–1	22 (59.5)	170 (82.1)	0.002
2–3	15 (40.5)	37 (17.9)	
**Type of cancer**			
Rectal cancer (Adenocarcinoma)	29 (78.4)	153 (73.9)	0.566
Anal cancer (Squamous cell carcinoma)	8 (21.6)	54 (26.1)	
**Distance from AV** (cm; mean ± SD)	7.2 ± 0.8	7.7 ± 1.8	0.169
**Stage according to AJCC 8th edition**			
2	4 (10.8)	91 (43.9)	<0.001
3	21 (56.8)	108 (52.2)	
4	12 (32.4)	8 (3.9)	
**Type of Therapy**			
LC-RT	10 (27.0)	131 (63.3)	0.016
TNT	15 (40.5)	66 (31.9)	
First line	12 (32.5)	10 (4.8)	
**Starting therapy from diagnosis** (days; mean ± SD)	38.3 ± 14.3	33.5 ± 14.9	0.083
Radical resection or resection not needed *^?^ (%)	21/34 * (61.7)	196/202 * (97)	0.021
**Time to radical resection** (months; mean ± SD)	7.8 ± 0.8	6.5 ± 0.3	0.187

Legend: ECOG-PS: Eastern Cooperative Oncology Group–Performance Status scale; NRS: Nutrition Risk Screening score; AV: anal verge; AJCC: American Joint Committee on Cancer; LC-RT: long course chemio-radiotherapy; TNT: Total Neoadjuvant Therapy. * 3 patients in the colostomy group and 5 patients in the control group still ongoing therapy; ^?^ 6 patients in the colostomy group and 50 patients in the control group did not need radical resection for complete clinical response after therapy.

## Data Availability

Data described in the manuscript, code book and analytic code will be made available upon reasonable request to the corresponding author.
